# TK2-related mitochondrial disorder is not restricted to the skeletal muscle

**DOI:** 10.1016/j.ymgmr.2018.06.003

**Published:** 2018-06-09

**Authors:** Josef Finsterer, Fulvio A. Scorza, Ana C. Fiorini, Antonio-Carlos G. de Almeida, Carla A. Scorza

**Affiliations:** a Krankenanstalt Rudolfstiftung, Messerli Institute, Veterinary University of Vienna, Vienna, Austria; b Disciplina de Neurociência, Escola Paulista de Medicina/Universidade Federal de São Paulo (EPM/UNIFESP), São Paulo, Brazil; c Programa de Estudos Pós-Graduado em Fonoaudiologia, Pontifícia Universidade Católica de São Paulo (PUC-SP), Departamento de Fonoaudiologia, Escola Paulista de Medicina/Universidade Federal de São Paulo (EPM/UNIFESP), São Paulo, Brazil; d Laboratório de Neurociência Experimental e Computacional, Departamento de Engenharia de Biossistemas, Universidade Federal de São João del-Rei (UFSJ), Minas Gerais, Brazil

**Keywords:** Mitochondrial, Myopathy, mtDNA depletion, Thymidine-kinase-2

With interest we read the article by Wang et al. about the clinical and molecular spectrum of human thymidine-kinase-2-(TK2)-related disease [1]. We have the following comments and concerns.

Though TK2 mutations predominantly manifest in the skeletal muscle, also the brain, eyes, heart, and liver can be affected [1]. Cerebral manifestations of TK2 mutations include cognitive impairment, epilepsy, loss of motor skills, developmental delay, motor development delay, feeding difficulties, muscle hypotonia, leucoencephalopathy, cerebral atrophy, cortical laminar necrosis, and cerebellar neuronal degeneration [[2], [3], [4]]. Since some of these manifestations may be also attributable to myopathy (e.g. hypotonia, respiratory insufficiency), it is often difficult to differentiate if a particular feature is attributable to affection of either the one or other organ, or both. Thus, we should know if all included patients underwent cerebral imaging to assess to which degree the phenotype was attributable to cerebral involvement.

So called “liver enzymes” may not only derive from the liver but may occur in other organs as well. Regarding transaminases GOT and GPT, and LDH, they are also produced in the muscle and may reflect rather muscle destruction than liver disease. Thus, we should be informed if there were morphological or structural liver abnormalities or other indications, such as hyperammonemia and liver failure [5], or death from liver failure, demonstrating that the liver was indeed an organ clinically affected in TK2-disease.

Recently, the phenotypic spectrum of TK2 mutations has been extended by the description of a female TK2 carrier manifesting with lethal hypertrophic cardiomyopathy [4]. Cardiac involvement became clinically apparent by the age of 13 months and progressed to severe heart failure by the age of 22 months [4]. It would be of value to mention if the 11 newly detected TK2 carriers were prospectively investigated for cardiac disease.

In summary, the genotypic and phenotypic spectrum of TK2 mutations is broader than so far anticipated.

## Conflict of interests

There are no conflicts of interest.

## Funding

No funding was received.

## Author contribution

All authors contributed equally (JF: design, literature search, discussion, first draft, FAS, ACF, A-CGA, CAS: literature search, discussion, critical comments).

## Ethical approval

The research has been given ethical approval.
